# Supplementary material for: Patterns of Intron Gain and Loss in Fungi
Source: PLoS Biol. 2004 Nov 30;2(12):e422. doi: 10.1371/journal.pbio.0020422 (PMC532390; doi:10.1371/journal.pbio.0020422)
Supplement: Table S1 — Also available at http://genes.mit.edu/NielsenEtAl/. (4.3 MB ZIP). [file pbio.0020422.st001.zip › NielsenEtAl/html/1278.html]

AN3846.1.NCU01271.1.MG02441.1.FG05903.1


```
 CLUSTAL W (1.82) Multiple Sequence Alignments - Introns Inserted


Sequence 1: MG02441.1	597 aa
Sequence 2: FG05903.1	448 aa
Sequence 3: NCU01271.1	484 aa
Sequence 4: AN3846.1	450 aa
Alignment Length: 609 aa
Number Identitical Residues: 227 aa
Alignment Score (without introns) 10833


MG02441.1 	MPTRSRSAACLLALLHVCMSTKALNHRILRTSKAGVTTTTCGLGIDCSPTPYTSRV1IGT
NCU01271.1	----------------MVMPTVVSDRMG-----------------TIDFIDYTNNH~VFS
FG05903.1 	----------------MVMATAVLDT-------------------------YNHNV~N--
AN3846.1  	----------------MVMAPTACVPAE-----------------VCMPKLYDK--~---
          	                : *.. .                            *        

MG02441.1 	1RTKQYSTDCLWSDQRQKHPPRLPGPCSSSPTKISATERPFTSSTLDLLLPLLKSPKQPE
NCU01271.1	~-----------------------------------------KCQTDSLNTVNN------
FG05903.1 	~-----------------HP------------------------HIALKKPIIR------
AN3846.1  	~----------------------------------------PDFYVNNIHQFSN------
          	                                         ..        . .      

MG02441.1 	LFLLDLHFDNHVPEFHPSHQLPHRTSSKALPLTEGESRKPRQ0KTTPGFLDHHHNGELVS
NCU01271.1	-----------------------------------GSLKHDD~YLHG-----LANGKLVA
FG05903.1 	-----------------------------------DSIEQHD~VITP-------------
AN3846.1  	-------------------------------------HGADD~LSQS---------RLAA
          	                                         :                 :

MG02441.1 	KAMIGGALRHRVESIDPEFCDAGDEDTFFVADLGHVYRQHLRWKKNLPRVKPFY-~-A1V
NCU01271.1	KQMIGDALRQRVESIDSEFCEPGDEDTFFVADLGEVYRQHLRWKLNLPRVKPFY-~-A1V
FG05903.1 	KQLIGQALHQRVEAIDHEMCEPGDEDTFFVADLGEVYRQHLRWKKNLPRVRPFY-~-A1V
AN3846.1  	KDLVLNVLKKRAAEVDTDRCAPGEEDAFYVADMGEVYRQHLRWKMNLSRVRPFYE1LA~V
          	* ::  .*::*.  :* : * .*:**:*:***:*.********* **.**:***.  * *

MG02441.1 	KCNPDPKVLRLLAELGNGFDCASKTEIKQVLDMGVDPSRIIYAQPCKTNSYVRYVAAEGV
NCU01271.1	KCHPDERLLQLLAALGTGFDCASKAEIEQVLRMGVDPSRIIYAQPCKTNSYLRYVAQQGV
FG05903.1 	KCNPDPQIIKLLSELGTGFDCASKTEIEQVLSAGLSPDRIIYAQPCKTNSYVRYVKSVGV
AN3846.1  	KCNPDPEVLRLMAKLGNGFDCASKAEIDMALSTGVDPSRIIYAQPCKTKSYLRHAAKVGV
          	**:** .:::*:: **.*******:**. .*  *:.*.**********:**:*:.   **

MG02441.1 	RQMTFDNADELYKIAKLFPEAELYLRILTDDSSSLCRLSLKFGASLDATDGLLALARELG
NCU01271.1	RQMTFDNADELRKIARLYPDAELFLRILTDDSSSLCRFSMKFGASLDSTDGLLGLARQLG
FG05903.1 	KQMTFDNADELYKIAKLYPGAELFLRIMTDDSESLCRFSMKFGAAMDTTEGLLALAKDLG
AN3846.1  	KQMTFDNADELYKIKACYPEAELYLRILTDDSNSLCQFSMKFGASLDVARQLLELAHQLE
          	:********** **   :* ***:***:****.***::*:****::* :  ** **::* 

MG02441.1 	LNVVGVSFHVGSGASDPLAFLKAVRDANFVFEQAAAHGFDMKTLDVGGGFCSDDTFEAMA
NCU01271.1	LNVVGVSFHVGSGASDPTAFLKAVQDAHVVFQQAAAYGYSLKTLDVGGGFCSDDSFEQMA
FG05903.1 	LNVVGVSFHVGSGASDPLAFFKAVRDAHTVFQQARDFGFAMRTLDVGGGFCG-DTFEAMA
AN3846.1  	LKIVGVSFHVGSGAEDPKAFLKAVQDARLVFDQAAEIGHELHTLDVGGGFTG-ETFEKFA
          	*::***********.** **:***:**. **:**   *. ::******** . ::** :*

MG02441.1 	GVLRDALDEYFPPHRGVSLIAEPGRYFVSAAFTIACNVIARRTVEDPN---------GSV
NCU01271.1	NVLRAALDEYFPAHTGVNLIAEPGRYYASSAFTLACNIIARRTIQDGSAVSVSDSSSMSD
FG05903.1 	NVLRGALDEFFPPHSGVEIIAEPGRYYVATAFTIACNIIARRTVEDP-----------TL
AN3846.1  	AVLDSALETYFPPN--IRIIAEPGRYYVGGAFTLAANIIARRGVQDPE----------NP
          	 **  **: :**.:  : :*******:.. ***:*.*:**** ::* .          . 

MG02441.1 	NGSLA-AAPSYMVYVNDGLYGNFSSIMFDHQHPVAKVLRSSGNFFYNTTDADSPESPSEG
NCU01271.1	DGSVNNGDARYMVYVNDGLYGNFSSIMFDHQHPVAKILRAGGRTMYNSVAAH-ESSAEDA
FG05903.1 	DG------KGYMLYVNDGVYGNFSNIMFDHQQPTAKILRTSGKTLFETAAAE-PTPVGEG
AN3846.1  	SK------DAYMIYLNDGVYGNFSNIIFDHQHPEARILSCAS-----EINGR---PVSED
          	.         **:*:***:*****.*:****:* *::*  ..        .    .  : 

MG02441.1 	IEYSIWGPTCDGIDRISESVRFSQTIDVGDWLYFEDMGAYTKCSATRFNGFSDAHDVIYV
NCU01271.1	IEYSIWGPTCDGIDRITESIRFREILDVGDWLYFEDMGAYTKCSATTFNGFSNEHDVIYV
FG05903.1 	FEYSIWGPTCDGIDRITDSTRFDPILDVGDWLYFEDMGAYTKCSATQFNGFSNAHDVIYV
AN3846.1  	VTYSIWGPTCDGIDVISQRSVLPGLLDVGDWLYFEEMGAYTRCSATRFNGFSDNHEVIYI
          	. ************ *::   :   :*********:*****:**** *****: *:***:

MG02441.1 	CSEPAARAMMLNYI
NCU01271.1	CSEPGAMALLGL--
FG05903.1 	CSEPGAKALLGL--
AN3846.1  	SSEAGASALLEY--
          	.**..* *::
```
